# Mechanism underlying the effect of Pulsatilla decoction in hepatocellular carcinoma treatment: a network pharmacology and in vitro analysis

**DOI:** 10.1186/s12906-023-04244-w

**Published:** 2023-11-10

**Authors:** Kuijie Liu, Zhenyu Cao, Siqi Huang, Fanhua Kong

**Affiliations:** 1 Department of General Surgery, The Second Xiangya Hospital of Central South University, Changsha, Hunan China; 2 Institute of Hepatobiliary Diseases of Wuhan University, Transplant Center of Wuhan University, National Quality Control Center for Donated Organ Procurement, Hubei Key Laboratory of Medical Technology on Transplantation, Hubei Clinical Research Center for Natural Polymer Biological Liver, Hubei Engineering Center of Natural Polymer-based Medical Materials, Zhongnan Hospital of Wuhan University, Wuhan, Hubei China; 3 Department of Integrated Traditional Chinese & Western Medicine, The Second Xiangya Hospital of Central South University, Changsha, Hunan China

**Keywords:** Hepatocellular carcinoma, Pulsatilla decoction, Network pharmacology, Traditional Chinese medicine, Proliferation, Apoptosis.

## Abstract

**Background:**

Currently, hepatocellular carcinoma (HCC) is associated with a poor prognosis. Moreover, there exist limited strategies for treating HCC. Pulsatilla decoction (PD), a traditional Chinese medicine formula, has been used to treat inflammatory bowel disease and several cancer types. Accordingly, we explored the mechanism of PD in HCC treatment via network pharmacology and in vitro experiments.

**Methods:**

Online databases were searched for gene data, active components, and potential target genes associated with HCC development. Subsequently, bioinformatics analysis was performed using protein–protein interaction and Network Construction and Kyoto Encyclopedia of Genes and Genomes (KEGG) to screen for potential anticancer components and therapeutic targets of PD. Finally, the effect of PD on HCC was further verified by in vitro experiments.

**Results:**

Network pharmacological analysis revealed that 65 compounds and 180 possible target genes were associated with the effect of PD on HCC. These included PI3K, AKT, NF-κB, FOS, and NFKBIA. KEGG analysis demonstrated that PD exerted its effect on HCC mainly via the PI3K-AKT, IL-17, and TNF signaling pathways. Cell viability and cell cycle experiments revealed that PD could significantly inhibit cancer cell proliferation and kill HCC cells by inducing apoptosis. Furthermore, western blotting confirmed that apoptosis was mediated primarily via the PI3K-AKT, IL-17, and TNF signaling pathways.

**Conclusion:**

To the best of our knowledge, this is the first study to elucidate the molecular mechanism and potential targets of PD in the treatment of HCC using network pharmacology.

**Supplementary Information:**

The online version contains supplementary material available at 10.1186/s12906-023-04244-w.

## Introduction

Hepatocellular carcinoma (HCC) is a highly prevalent cancer globally and is associated with poor prognosis, owing to which it has resulted in a considerable economic burden [1, 2]. Currently, HCC is the second leading cause of death among malignant tumors, with the 5-year survival rate of HCC being extremely low [3, 4]. The primary treatment strategy for early HCC is surgical treatment, and there exist no effective strategies for treating advanced HCC [5, 6]. In recent years, with the advent of targeted therapy and immunotherapy, the prognosis of HCC has improved significantly. For example, immunotherapy has shown encouraging findings in the treatment of HCC [7]. However, immune escape and the emergence of multidrug resistance have limited the application of immunotherapy in HCC. Thus, further research is required to develop new therapeutic agents and targets.

Traditional Chinese medicine (TCM) compounds contain a variety of active antitumor components, owing to which these compounds have been shown to improve the prognosis of patients with several cancer types and have lower side effects than conventional treatments [8]. TCM compounds can inhibit the proliferation, invasion, metastasis, and angiogenesis of tumor cells. In addition, they can enhance the anti-tumor ability of the body and enhance the efficacy of other conventional drugs administered in combination [9]. Pulsatilla decoction (PD) is a TCM compound consisting of *Radix Pulsatillae*, *Cortex Phellodendri*, *Rhizoma Coptidis*, and *Cortex Fraxini* and is currently used to treat colitis and intestinal tumors [10–12]. A few studies have also assessed the application of PD in the treatment of malignant tumors. One such study demonstrated that PD can enhance the antitumor effect of colon cancer by enhancing tumor sensitivity to 5-FU [10]. Another study by Wu et al. revealed that PD can inhibit the proliferation of pancreatic cancer cells as well as induce apoptosis, as assessed using the cell counting kit (CCK-8) and via animal experiments [13]. In addition, PD may exert anticancer effects in colon cancer via multiple components, targets, and pathways, as determined using network pharmacology [14]. *Rhizoma Coptidis*, one of the main components of PD, has been proven to induce inhibitory and lethal effects on HCC cells [15–17]. However, the antitumor mechanism of PD, especially HCC, is still unknown.

In recent years, bioinformatic studies of large online databases provide an ideal platform for researchers to study the active components and therapeutic targets of TCM compounds. This will facilitate the further discovery of new active antitumor components and new therapeutic targets [18]. Network pharmacology is another research technique that is conducive to the development of new therapeutic targets and strategies. It functions by elucidating the relationship between drug, gene, and tumor as well as identifying the relationship between active drug ingredients and target genes [19]. More importantly, network pharmacology can be used to enhance the efficacy of antitumor drugs and reduce the side effects by analyzing the occurrence and development of diseases as well as identifying disease regulatory pathways [20].

This study aimed to assess the efficacy of PD in the treatment of HCC as well as identify the pathways involved in the antitumor effect of PD, using network pharmacology.

## Materials and methods

### Data collection

#### Composition of PD

PD is composed of *Radix Pulsatillae*, *Cortex Phellodendri*, *Rhizoma Coptidis*, and *Cortex Fraxini*. The active compounds of PD, with an oral bioavailability (OB) of ≥ 30% and drug-likeness (DL) of ≥ 0.18, were screened from the Chinese Medicine Systematic Pharmacology Database and Analysis Platform (TCMSP: https://old.tcmsp-e.com/tcmsp.php) and Chinese Medicine Molecular Mechanism Biological Information Analysis Tool (http://bionet.ncpsb.org.cn/batman-tcm/).

#### Predicted targets of PD and HCC-related genes

The putative targets of the four components of PD were collected from TCMSP and DRUGBANK (https://go.drugbank.com/), after that input all putative targets into the uniport database (https://www.uniprot.org/) to transfer corresponding gene symbols of the target proteins. The hepatocellular carcinoma as key term search for two GeneCards (https://www.genecards.org/, ver, 5.11) and OMIM (http://www.omim.org/; updated: July 15, 2022) databases to acquire HCC-related targets. Based on results above two steps, a Venn diagram was drawn to exhibit the intersection targets of PD and HCC. The intersection targets of PD and HCC as hub gene for subsequent study.

### Bioinformatics analysis

#### Protein–protein interaction (PPI) analysis

In order to reveal relationship between targets and identify core target, the PPI analysis were performed. The overlapping targets of compounds in PD and HCC, as hub genes, was entered into the online STRING database (https://cn.string-db.org/) for PPI analysis. The species was limited to Homo sapiens, and the interaction score was retained as > 0.990, hidden disconnect targets. After that, the TSV files, which containing results of PPI analysis were analyzed using the STRING tool, was download. Subsequently, the TSV files was put into the Cytoscape 3.7.1 to construct and visualize the PPI network. The CytoNCA plugin in the Cytoscape software were used to calculate degree, that a key topological parameter characterizes the most significant nodes in the network. Higher degree quantitative values of targets, more crucial in PD for treating HCC.

#### Gene ontology (GO) and Kyoto Encyclopedia of Genes and Genomes (KEGG) enrichment analyses

After transferring official gene symbols of hub genes to related Entrez IDs, Gene Ontology (GO) enrichment analysis and Kyoto Encyclopedia of Genes and Genomes (KEGG) pathway enrichment analysis were conducted based on R 3.6.3 and plugins in the R-package such as clusterProfiler, ggplot2, enrichplot, pathview, colorspace, DOSE, org.Hs.eg.db, etc.). Only GO functional and KEGG pathway term with p value < 0.05 were identified statistically significant and stayed for subsequent study.

#### Network construction

The network construction is aim to clearly elucidated relationship between nodes. In this study, three network constructions were established and visualized through Cytoscape 3.7.1. (1) bioactive component-disease target network (C-D-T) describe relationship among ingredients, common targets, and disease. The node shape and color represent ingredients, targets, and disease, respectively, while edges stand for interactions between nodes. The network elucidates the interaction between the active components of PD and the targets anti-HCC. (2) PPI network and (3) Bioactive ingredient-pathway-target network. The bioactive ingredient, top 20 signal pathways with the smallest p value of KEGG enrichment analysis, and the corresponding targets of each pathway were submitted into Ctyoscape3.7.1, and drawn bioactive ingredient-pathway-target network diagram of PD against HCC.

#### Molecular docking

**Fig. 1:**
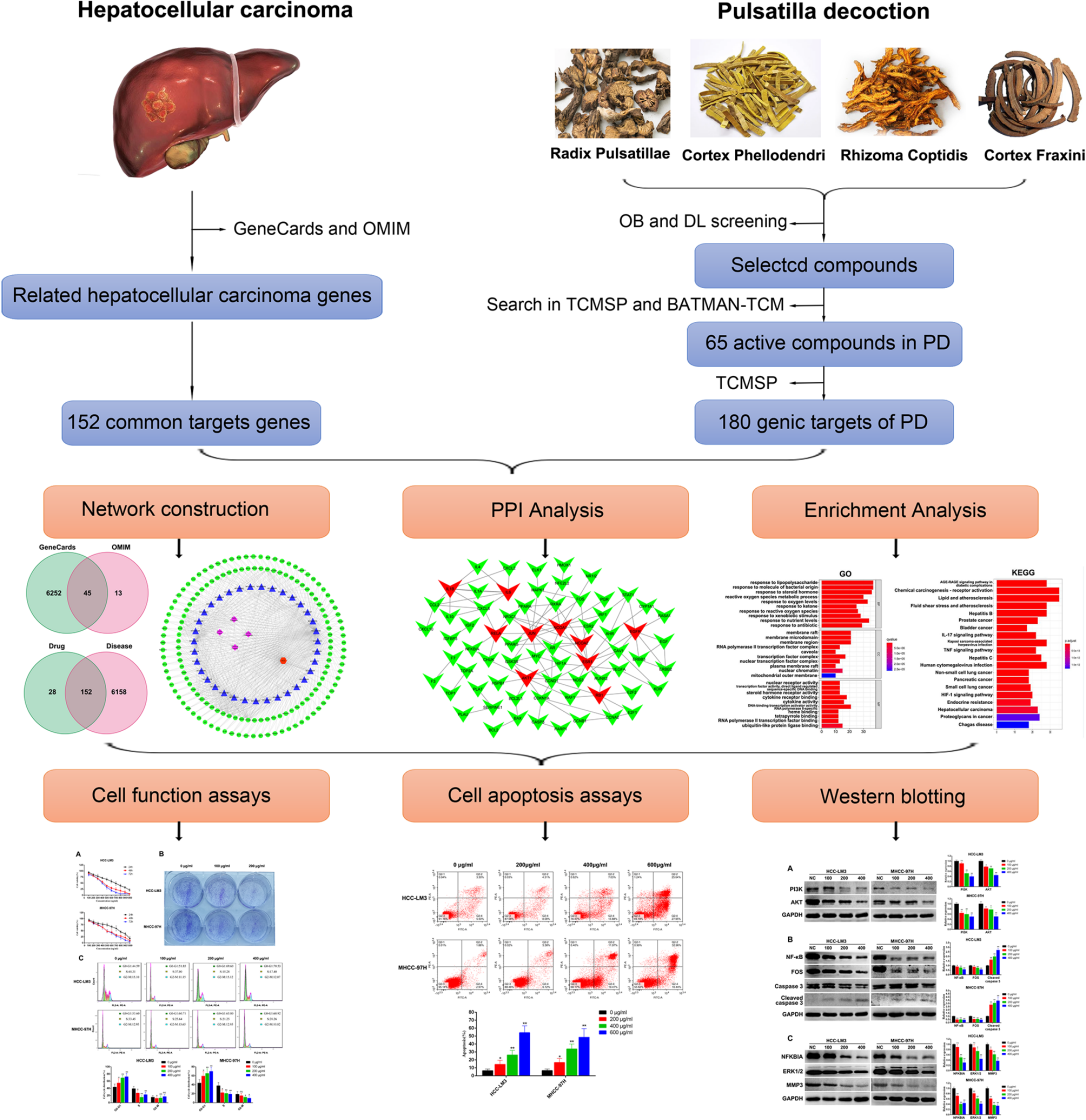
The technical strategy used in this study. The active components and potential target genes of PD were analyzed by network pharmacology. Network structure, PPI and enrichment analysis were further analyzed. Finally, in vitro experiments were conducted to verify the therapeutic effect of PD on HCC and explore its potential molecular mechanism

Molecular docking is widely used for network pharmacology as it can precisely predict the conformation of small-molecule ligands within the target binding site and assess the binding affinity. For molecular docking, candidate target proteins and compounds were selected based on the results of the PPI network, C-D-T network, and KEGG pathway analyses. Molecular docking was used for validating the compound–target interaction using the Molecular Operating Environment (MOE) (v2015.10) software, as follows. Firstly, the 3D structure of the candidate protein was downloaded from Protein Data Bank (PDB) (http://www.rcsb.org/) with the species limited to “Homo sapiens.” The structure of the compound was obtained from PubChem (https://pubchem.ncbi.nlm.nih.gov/). Subsequently, the 3D structure of the candidate protein was imputed into the MOE to construct a mating pocket after deleting water molecules, preparing the protein structure, and minimizing energy. Lastly, the mating pocket was docked with the candidate compound. Figure 1 shows the experimental methods used in this research.

### Experimental validation

#### Preparation of PD extract

*Radix Pulsatillae* (Bai Tou Weng, *Pulsatilla chinensis* (Bge.) Regel), *Cortex Phellodendri* (Huang Bai, *Phellodendron amurense* Rupr.), *Rhizoma Coptidis* (Huang Lian, *Coptis chinensis* Franch.), and *Cortex Fraxini* (Qin Pi, *Fraxinus chinensis* Roxb.) were obtained from the Second Xiangya Hospital of Central South University and were used to prepare PD aqueous extract, as per the Chinese Pharmacopoeia (2020 edition) [21]. Briefly, 20 g of *Radix Pulsatillae*, 16 g of *Cortex Phellodendri*, 8 g of *Rhizoma Coptidis*, and 16 g of *Cortex Fraxini* were soaked with twice the volume of pure water for 30 min and then boiled for 1.5 h. After cooling to room temperature, the mixture was centrifuged at 10,000 rpm for 30 min to collect the supernatant, and the process was repeated twice. The collected supernatant was vacuum dried to obtain a powder. Then, the powder was solubilized with dimethyl sulfoxide (DMSO; 200 mg/ml), filtered, and stored at − 20℃ until further use.

#### Cell culture

HCC-LM3 and MHCC-97 H cell lines were purchased from the Institute of Biochemistry and Cell Biology, Chinese Academy of Sciences. HCC cell lines were cultured in Dulbecco’s Modified Eagle Medium supplemented with 10% fetal bovine serum and cultured in Petri dishes at 37 °C with 5% CO_2_ and 95% air.

#### Cell viability assay

After cell counting, HCC-LM3 and MHCC-97 H cells (logarithmic growth stage) were seeded in 96-well plates at 10,000 cells/well. After incubation for 24 h, the cells were treated with different concentrations of PD for 24, 48, and 72 h. Then, all cells were treated with 0.5% DMSO for 24, 48, and 72 h. Then, the fresh medium was replaced and 10 µl of CCK-8 (Genview, USA) reagent was added to each well. After 2 h incubation, cell viability in each well was determined using a spectrophotometer.

#### Colony formation assay

After cell counting, HCC-LM3 and MHCC-97 H cells with a cell confluence of 70–80% were seeded in 6-well plates with 3000 cells/well and incubated for 24 h at 37 °C. Based on the experimental results of the cell viability assay. Subsequently, 100 and 200 µg/ml of PD were added to the 6-well plates and incubated for 2 weeks at 37 °C with 5% CO_2_ and 95% air. Then, the medium was removed, and 4% paraformaldehyde was added and fixed for 30 min. The samples were then stained with crystal violet for 2 h, cleaned, and then photographed for analysis.

#### Flow cytometry for cell cycle analysis

After cell counting, HCC-LM3 and MHCC-97 H cells in the logarithmic stage of growth (cell confluence: 70–80%) were seeded in 6-well plates at 3 × 10^5^ cells/well and incubated at 37℃ for 24 h. Based on the experimental results of the cell viability assay. Subsequently, 100, 200, and 400 µg/ml of PD were added to the 6-well plates. After 48 h, the cells were harvested and fixed with 70% ethanol overnight. The fixed cells were then stained with propidium iodide (PI, 50 µg/ml; Genview, USA) for 45 min and were analyzed using a Canto II flow cytometer (BD Bioscience, USA).

#### Flow cytometry analysis of cell apoptosis

HCC-LM3 and MHCC-97 H cells in the logarithmic growth stage were seeded in 6-well plates at 3 × 10^5^ cells/well and incubated at 37℃ for 24 h. Based on the experimental results of the cell viability assay. Subsequently, 200, 400, and 600 µg/ml of PD were added to the 6-well plates for 48 h. Then, cells were digested with trypsin without EDTA. The harvested cells were then resuspended in 200 µl 1× binding buffer and incubated for 15 min with 5 µl Annexin V-FITC and 5 µl PI (Genview, USA). The cells were then analyzed using a Canto II flow cytometer (BD Bioscience, USA). The obtained data were analyzed using the FlowJo ver.7.6 Software (De Novo Software, USA).

#### Western blotting

After cell counting, HCC-LM3 and MHCC-97 H cells in the logarithmic growth stage (cell confluence: 70–80%) were seeded in 6-well plates at 3 × 10^5^ cells/well and incubated for 37℃ for 24 h. PD (100, 200, and 400 µg /ml) was then added to the plates and treated for 48 h. The protein was denatured, electrophoresed, and transferred to the PVDF membrane. The PVDF membrane was then blocked with 5% BSA for 2 h at room temperature. And the blots were cut prior to hybridisation with antibodies during blotting. Subsequently, antibodies were added and incubated overnight at 4 °C. The primary antibodies used were those against PI3K (1:1000, ABclonal, Cat No: A0265), AKT (1:1000, ABclonal, Cat No: A17909), NF-κB (1:1000, ABclonal, Cat No: A6667), FOS (1:1000, Proteintech, Cat No: 66590-1-Ig), Caspase 3 (1:1000, Proteintech, Cat No: 19677-1-AP), NFKBIA (1:1000, ABclonal, Cat No: A11397), ERK1/2 (1:1000, Proteintech, Cat No: 16443-1-AP), MMP3 (1:1000, Proteintech, Cat No: 17873-1-AP), and GAPDH (1:10000, Proteintech, Cat No: 60004-1-Ig). The membrane was then incubated with the secondary antibody (1:5000, ABclonal) for 1 h. The enhanced chemiluminescence detection kit (Genview, USA) was used to detect the fluorescence of the bands, which were quantitatively analyzed using the ImageJ software (Version 11).

#### Ethics approval

This project was approved by the Medical Ethics Committee of the Second Xiangya Hospital of Central South University.

#### Statistical analysis

The student’s *t-test* and one-way ANOVA analysis were used for statistically analyzing all data, and the GraphPad Prism 6 software (La Jolla, CA, USA) was used for data processing. All data were expressed as means ± standard deviations, and results with P < 0.05 were considered statistically significant.

## Results

### Compounds and putative targets of PD

PD consists of *Radix Pulsatillae*, *Cortex Phellodendri*, *Rhizoma Coptidis*, and *Cortex Fraxini*. A total of 266 compounds were authenticated in PD, including 57 in *Radix Pulsatillae*, 140 in *Cortex Phellodendri*, 48 in *Rhizoma Coptidis*, and 21 in *Cortex Fraxini*. Based on the OB of ≥ 30% and DL of ≥ 0.18, 65 bioactive compounds (11 in *Radix Pulsatillae*, 37 in *Cortex Phellodendri*, 14 in *Rhizoma Coptidis*, and 3 in *Cortex Fraxini*) were examined further (Table 1).

**Table 1:** Compounds of pulsatilla decoction

MOL ID	MOL Name	OB	DL	Medicine
MOL006710	8-(beta-D-Glucopyranosyloxy)-7-hydroxy-6-methoxy-2 H-1-benzopyran-2-one	36.76	0.42	Cortex Fraxini
MOL006709	AIDS214634	92.43	0.55	Cortex Fraxini
MOL000358	beta-sitosterol	36.91	0.75	Cortex Fraxini, Radix Pulsatillae, Cortex Phellodendri
MOL001455	(S)-Canadine	53.83	0.77	Cortex Phellodendri
MOL005438	campesterol	37.58	0.71	Cortex Phellodendri
MOL002671	Candletoxin A	31.81	0.69	Cortex Phellodendri
MOL002670	Cavidine	35.64	0.81	Cortex Phellodendri
MOL002666	Chelerythrine	34.18	0.78	Cortex Phellodendri
MOL002651	Dehydrotanshinone II A	43.76	0.4	Cortex Phellodendri
MOL002643	delta 7-stigmastenol	37.42	0.75	Cortex Phellodendri
MOL002652	delta7-Dehydrosophoramine	54.45	0.25	Cortex Phellodendri
MOL002656	dihydroniloticin	36.43	0.81	Cortex Phellodendri
MOL006392	dihydroniloticin	36.43	0.82	Cortex Phellodendri
MOL000787	Fumarine	59.26	0.83	Cortex Phellodendri
MOL002672	Hericenone H	39	0.63	Cortex Phellodendri
MOL002673	Hispidone	36.18	0.83	Cortex Phellodendri
MOL000790	Isocorypalmine	35.77	0.59	Cortex Phellodendri
MOL002636	Kihadalactone A	34.21	0.82	Cortex Phellodendri
MOL002659	kihadanin A	31.6	0.7	Cortex Phellodendri
MOL006401	melianone	40.53	0.78	Cortex Phellodendri
MOL002660	niloticin	41.41	0.82	Cortex Phellodendri
MOL001131	phellamurin_qt	56.6	0.39	Cortex Phellodendri
MOL002641	Phellavin_qt	35.86	0.44	Cortex Phellodendri
MOL006413	phellochin	35.41	0.82	Cortex Phellodendri
MOL002644	Phellopterin	40.19	0.28	Cortex Phellodendri
MOL001771	poriferast-5-en-3beta-ol	36.91	0.75	Cortex Phellodendri
MOL002662	rutaecarpine	40.3	0.6	Cortex Phellodendri
MOL002663	Skimmianin	40.14	0.2	Cortex Phellodendri
MOL006422	thalifendine	44.41	0.73	Cortex Phellodendri
MOL001454	berberine	36.86	0.78	Cortex Phellodendri, Rhizoma Coptidis
MOL002894	berberrubine	35.74	0.73	Cortex Phellodendri, Rhizoma Coptidis
MOL001458	coptisine	30.67	0.86	Cortex Phellodendri, Rhizoma Coptidis
MOL000622	Magnograndiolide	63.71	0.19	Cortex Phellodendri, Rhizoma Coptidis
MOL013352	Obacunone	43.29	0.77	Cortex Phellodendri, Rhizoma Coptidis
MOL000785	palmatine	64.6	0.65	Cortex Phellodendri, Rhizoma Coptidis
MOL000762	Palmidin A	35.36	0.65	Cortex Phellodendri, Rhizoma Coptidis
MOL000098	quercetin	46.43	0.28	Cortex Phellodendri, Rhizoma Coptidis
MOL002668	Worenine	45.83	0.87	Cortex Phellodendri, Rhizoma Coptidis
MOL001984	3beta,23-Dihydroxy-lup-20(29)-ene-28-O-alpha-L-rhamnopyranosyl-(1–4)-beta-D-glucopyranosyl(1–6)-beta-D-glucopyranoside_qt	37.59	0.79	Radix Pulsatillae
MOL001978	Aureusidin	53.42	0.24	Radix Pulsatillae
MOL000354	isorhamnetin	49.6	0.31	Radix Pulsatillae
MOL001979	LAN	42.12	0.75	Radix Pulsatillae
MOL000211	Mairin	55.38	0.78	Radix Pulsatillae
MOL001971	Pulchinenoside C_qt	37.79	0.76	Radix Pulsatillae
MOL001973	Sitosteryl acetate	40.39	0.85	Radix Pulsatillae
MOL001985	ZINC01615307	56.38	0.87	Radix Pulsatillae
MOL001987	β-sitosterol	33.94	0.7	Radix Pulsatillae
MOL000449	Stigmasterol	43.83	0.76	Radix Pulsatillae, Cortex Phellodendri
MOL002903	(R)-Canadine	55.37	0.77	Rhizoma Coptidis
MOL002904	Berlambine	36.68	0.82	Rhizoma Coptidis
MOL002907	Corchoroside A_qt	104.95	0.78	Rhizoma Coptidis
MOL002897	epiberberine	43.09	0.78	Rhizoma Coptidis
MOL008647	Moupinamide	86.71	0.26	Rhizoma Coptidis

### Prediction targets and C-D-T network analysis

**Fig. 2:**
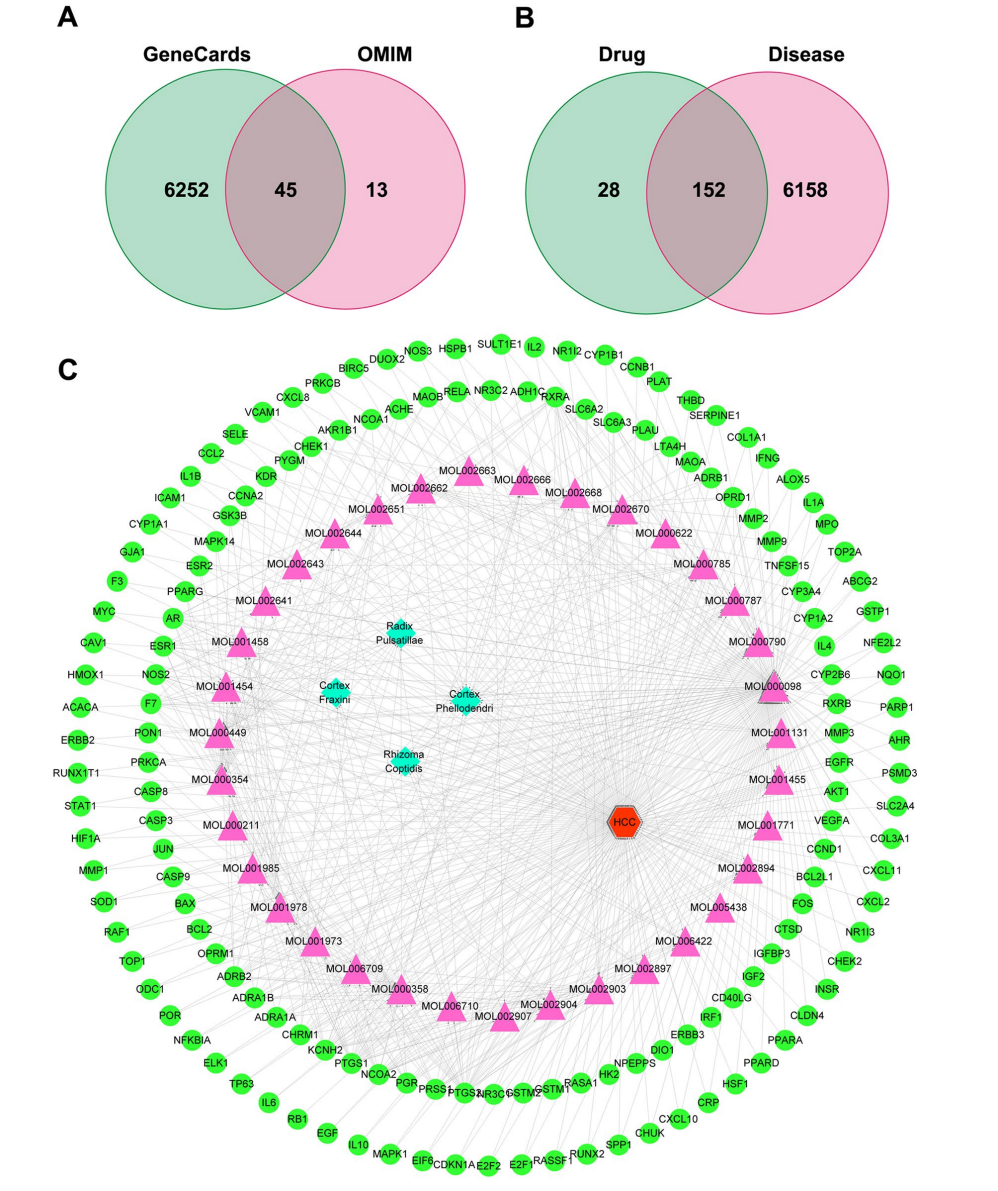
Prediction targets and C-D-T network analysis. (**A**) 6310 HCC-related genes. (**B**) 152 overlapping genes of compounds and HCC. (**C**) The C-D-T network

In total, 180 target genes from the TCSP and DRUGBANK databases were screened. Furthermore, using “HCC” as a term for research, 6297 related genes were retrieved from GeneCards with a relevance score of ≥ 1 and 58 related genes were obtained from OMIM. Eventually, 6310 HCC-related genes were obtained from both GeneCards and OMIM (Fig. 2A). Comparing the 180 putative target genes of the compounds with the 6310 HCC-related genes, 152 common genes were identified as hub genes for subsequent study (Fig. 2B).

As shown in Fig. 2C, the C-D-T network comprised 192 nodes and 585 edges. The green circle represents genes, the pink triangle represents compounds, the blue diamond represents the four herbs of PD, and the red hexagon represents HCC. The top three compounds with the highest degree were quercetin (MOL000098 127), stigmasterol (MOL000449, 22), and isorhamnetin (MOL000354, 21), which may play key roles in mediating the anticancer effect in HCC.

### Bioinformatics analysis

#### PPI network analysis

**Fig. 3:**
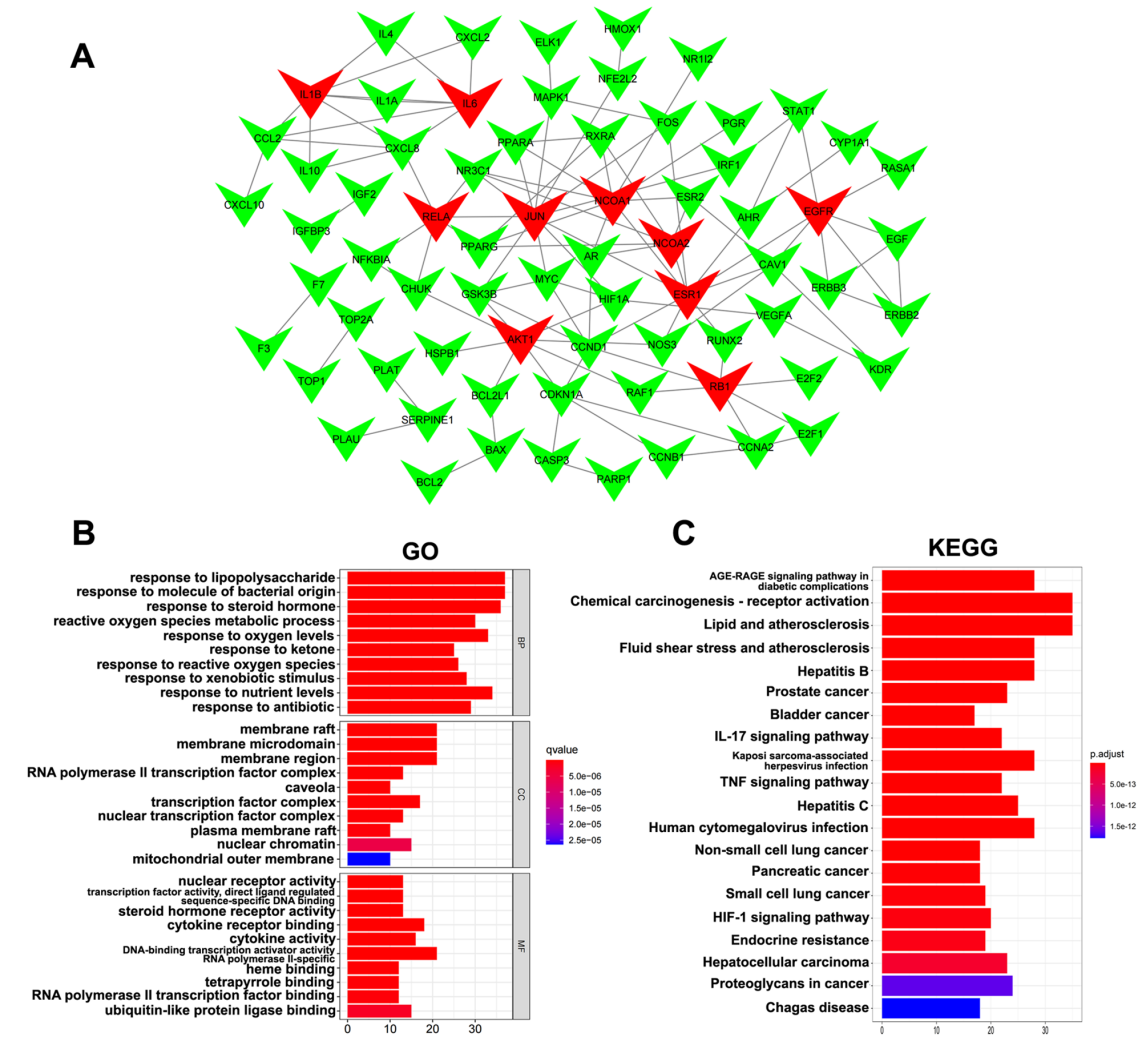
PPI network, GO and KEGG enrichment analysis. (**A**) PPI network for PD treating HCC. Red nodes stand for ranking top ten with degree for PD anti-HCC targets. (**B**) BP, CC, and MF analysis. The lower q-value and redder color indicated greater enrichment of the GO terms. (**C**) Top 20 KEGG enrichment pathways. The x-axis represents the counts target genes in each pathway and the ordinate represents pathway. Redder means lower p-value

To elucidate the interaction between HCC and PD-related targets, we analyzed the interaction between the two by establishing a PPI network. In addition, we analyzed 152 PD therapeutic targets using the STRING database, and the obtained data were analyzed by Cytoscape 3.7.1. As shown in Fig. 3A, the constructed PPI network contains 70 nodes and 109 edges. Table 2 lists the top 10 intensity data of the red nodes, which indicates that PD plays an important role in the treatment of HCC.

**Table 2:** The top 10 genes in the PPI network for degree nodes

NO.	Gene Name	Degree
1	JUN	11.0
2	ESR1	10.0
3	NCOA1	9.0
4	AKT1	8.0
5	IL1B	7.0
6	EGFR	7.0
7	RELA	6.0
8	EB1	6.0
9	IL6	6.0
10	NCOA2	6.0

#### GO enrichment analysis

The GO and KEGG analyses were used to reveal functions and pathways related to the 152 anti-HCC PD-related target genes. In total, 2508 GO terms were found to be associated with the 152 anti-HCC PD target genes. Among these, 2281 terms were related to biological process, 67 to cell composition, and 160 terms to molecular function. After screening the top ten most obvious differences in GO analysis, the targets of PD were found mainly located in the membrane raft and membrane microdomain and were characterized by nuclear receptor activity. In addition, these targets of PD were involved in response to lipopolysaccharides, molecules of bacterial origin, a steroid hormone, the metabolic process of reactive oxygen species, and oxygen levels for the treatment of HCC (Fig. 3B).

#### C-P-T and KEGG enrichment analyses

**Fig. 4:**
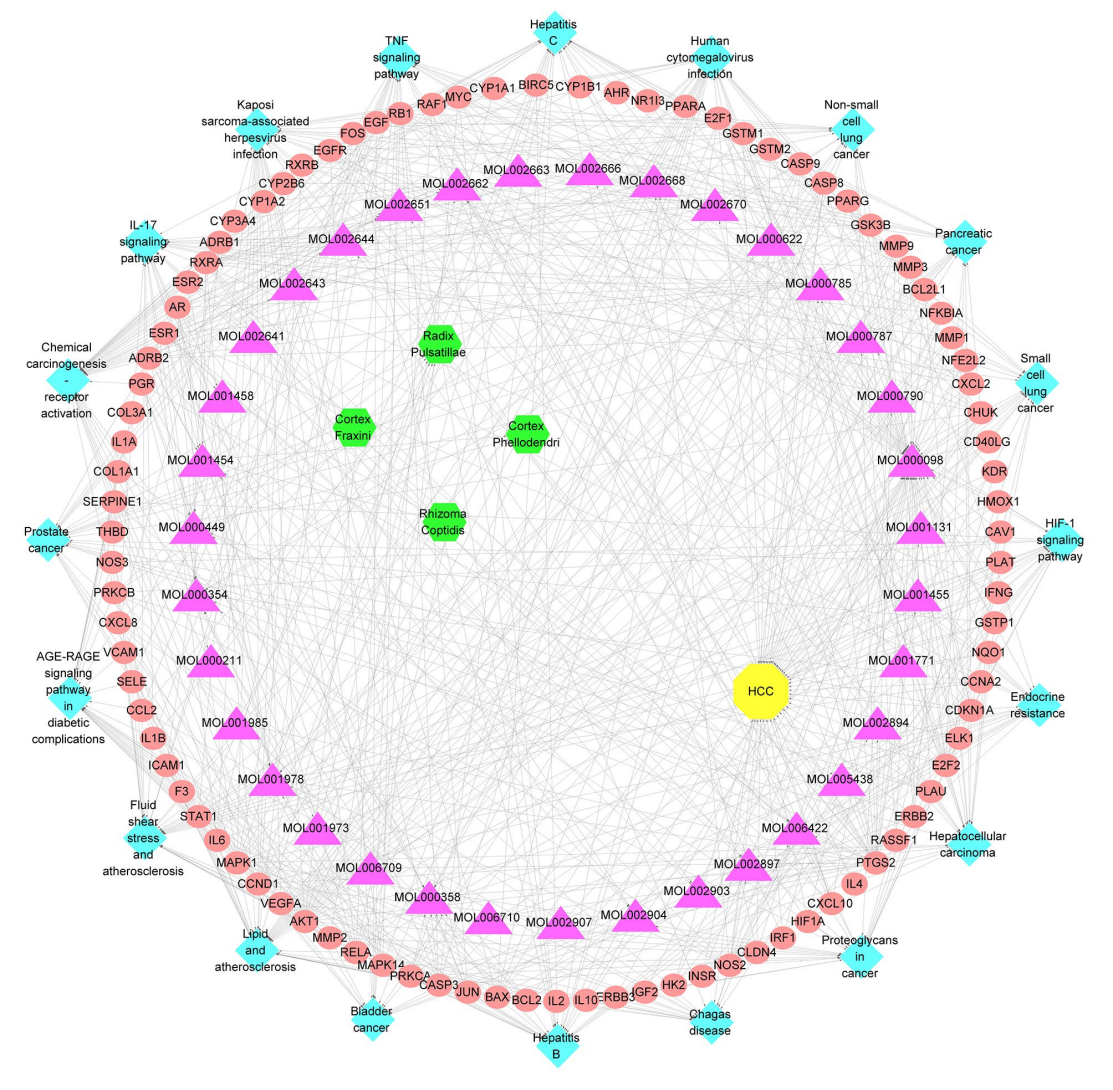
C-P-T network of top 20 pathway. The C-P-T network contains 153 nodes and 807 edges

To clarify the pathways associated with the anti-HCC effect of PD-related target genes, KEGG pathway analysis was performed. The results demonstrated that 145 of the 152 target genes were enriched in 168 pathways (P < 0.05). This indicated that the anti-HCC mechanism of PD could involve multiple target genes and pathways. The top 20 KEGG pathways with lower p values were visualized using a bar plot (Fig. 3C). Among the top 20 pathways, the TNF signaling pathway with a lower p-value was found to have a high association with carcinoma and was selected for subsequent validation (Supplementary Fig. 1). Besides, to elucidate the interrelationship among compounds, pathways, and targets of the top 20 pathways, the C-P-T network was constructed (Fig. 4), which included 154 nodes and 685 edges. The red circle indicates genes, the pink triangle indicates compounds, the blue diamond indicates the pathways, the green hexagon indicates the four herbs of PD, and the yellow octagon indicates HCC.

#### Molecular docking analysis

**Fig. 5:**
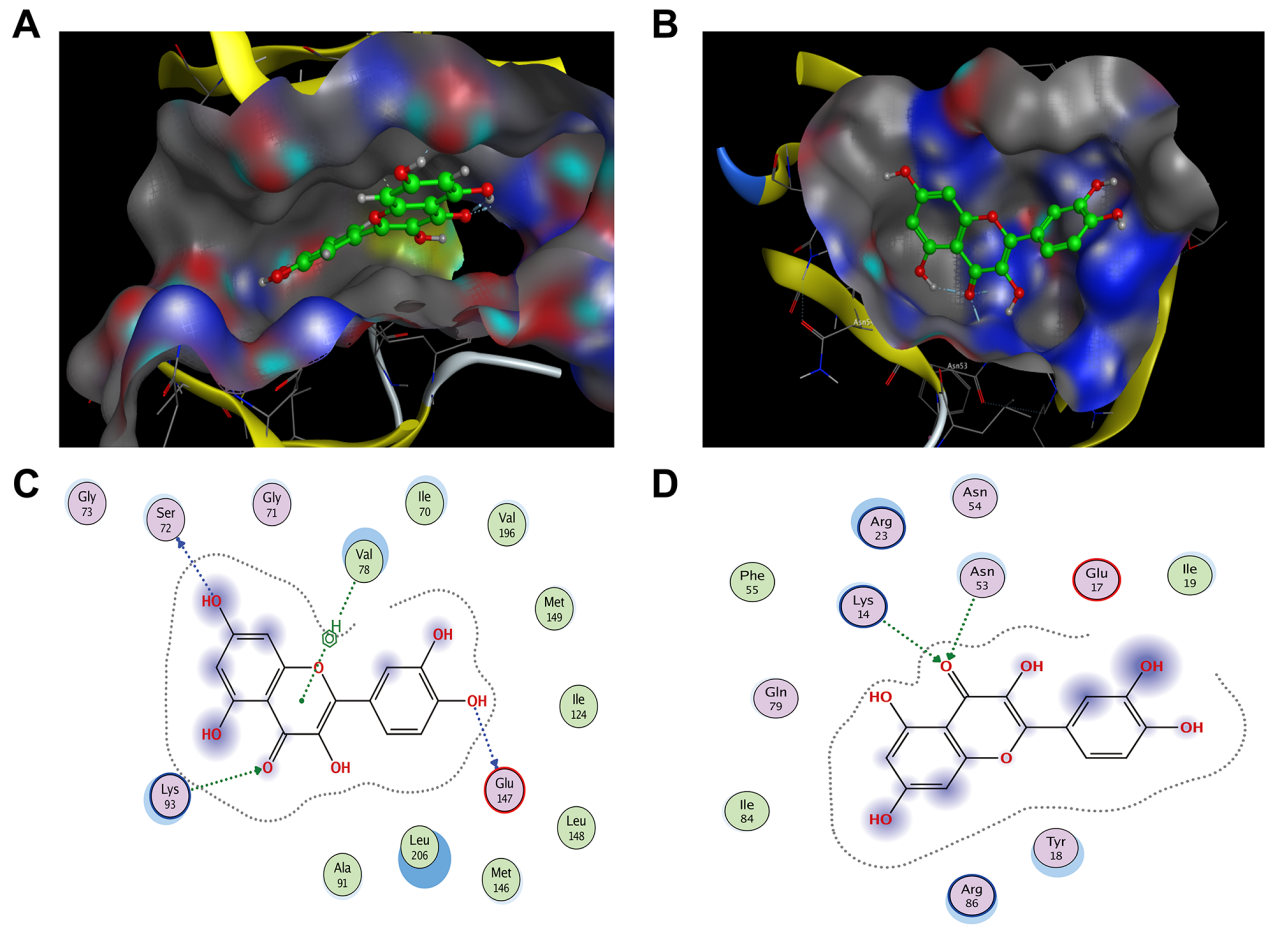
The docking model of quercetin with JUN and AKT1, respectively. Binding model of quercetin on the molecular surface of JUN (**A**), AKT1 (**B**). The interaction model of quercetin with JUN (**C**) and AKT1 (**D**). The ligands in binding model and interaction model are colored in green

Combining the results of PPI, C-D-T network analysis, and the TNF signaling pathway, the targets JUN (PDB code:2P33) and AKT1 (PDB code:1h10) with a higher degree were selected for docking with quercetin. The results of quercetin docking on JUN and AKT1 are shown in Fig. 5A, B. Quercetin is linked via four hydrogen bonds and one H-π bond with Ser72, Gly147, Lys93, and Val78 in JUN, (Fig. 5C), whereas it formed two hydrogen bonds with Lys14 and Asn53 in AKT1 (Fig. 5D).

In summary, the results of molecular docking illustrated that compounds and protein targets interacted well via different bonds. The matching degree between compounds and target proteins assessed using binding energy revealed that the lower the binding energy, the greater the stability (Table 3). This suggested that compounds could bind well with the active site of protein targets.

**Table 3:** Binding energy results of molecular docking (Kcal/Mol).

Ligands Receptors	JUN	AKT1
Quercetin	-7.4788	-5.2698

### PD inhibits HCC cell proliferation

**Fig. 6:**
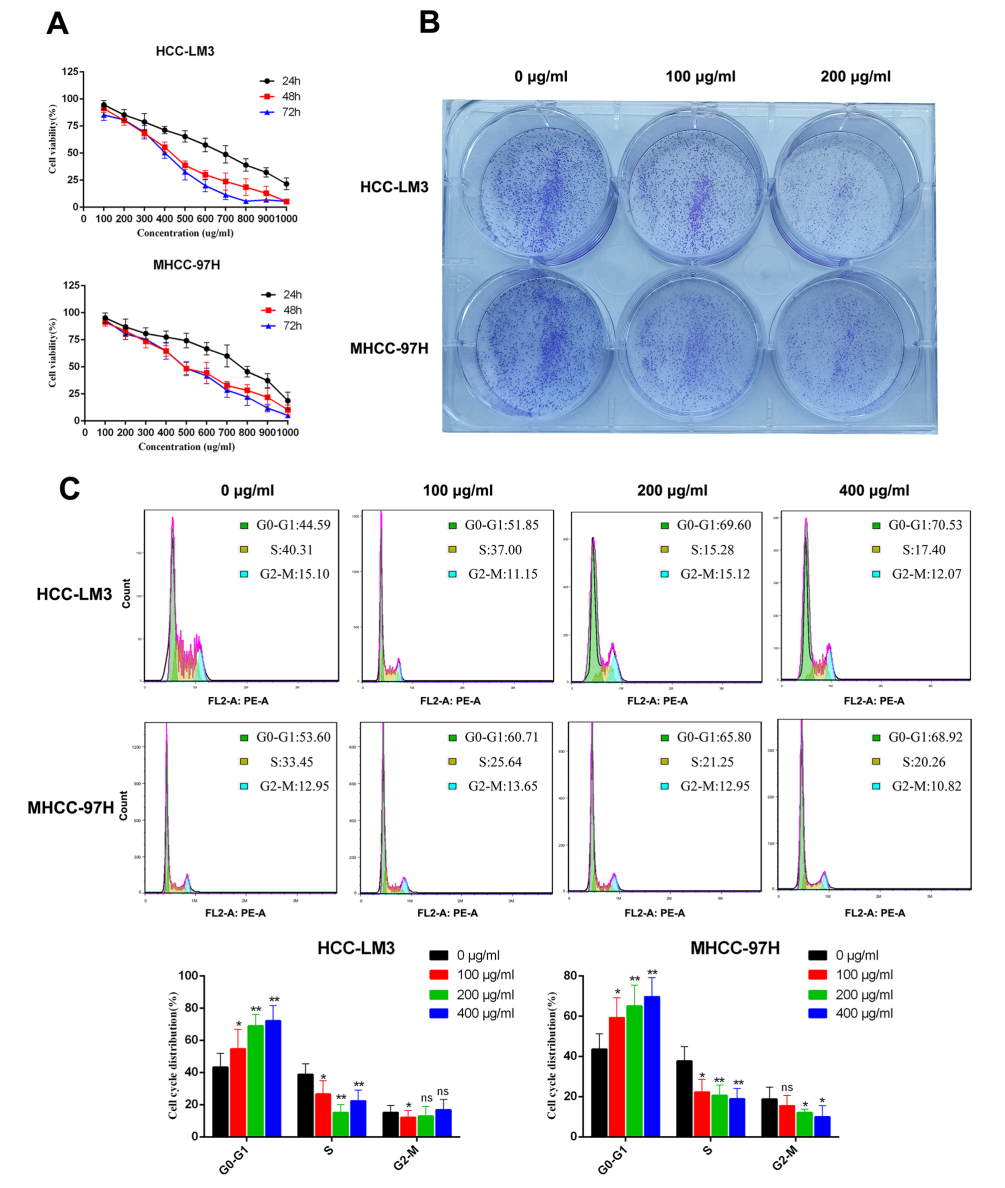
PD inhibits the proliferation of HCC cells. (**A**) Cell viability assay showed that PD could decrease the viability of HCC cells. The HCC cells were treated with PD at different concentrations for 24, 48 and 72 h. (**B**) Representative images showing colonies formed by HCC cells treated with various concentrations of PD for 2 weeks. (**C**) Representative images and statistical graphs of HCC-LM3 and MHCC-97 H cell cycle analysis. *P < 0.05, **P < 0.01 versus the untreated group

HCC-LM3 and MHCC-97 H cells were used to analyze the inhibitory effect of PD on HCC proliferation. Different concentrations of PD (100–1000 µg/ml) were tested for different incubation periods (24, 48, and 72 h). The DMSO toxicity assay confirmed that at the maximum concentration of 0.5%, DMSO did not exert a significant cytotoxic effect on HCC-LM3 and MHCC-97 H cells (Supplementary Fig. 2A). The survival rate of HCC cells decreased with the dose and exposure time (Fig. 6A). The half inhibitory concentration of PD at 24, 48, and 72 h is shown in Table 4. Cell viability analysis showed that PD had a significant killing effect on HCC cells, and the effect was stronger after 48 and 72 h of incubation than that after 24 h, whereas there was no significant difference between the effect at 48 and 72 h. For the colony formation assay, HCC-LM3 and MHCC-97 H cells were treated with concentrations of 0,100, and 200 µg/ml (Fig. 6B). The results revealed that PD significantly reduced colony formation in both cell types and could inhibit the proliferation of HCC cells. In addition, cell cycle analysis revealed a delayed G1/S transition and cycle arrest in HCC-LM3 and MHCC-97 H cells following treatment with PD (Fig. 6C). Therefore, PD can effectively inhibit the occurrence and development of liver cancer.

**Table 4:** The IC_50_ values of HCC cells treated with PD(µg/ml)

Cell lines	24 h	48 h	72 h
HCC-LM3	694.7	307.8	299.4
MHCC-97 H	701.9	360.1	386.1

### PD promotes HCC cell apoptosis

**Fig. 7:**
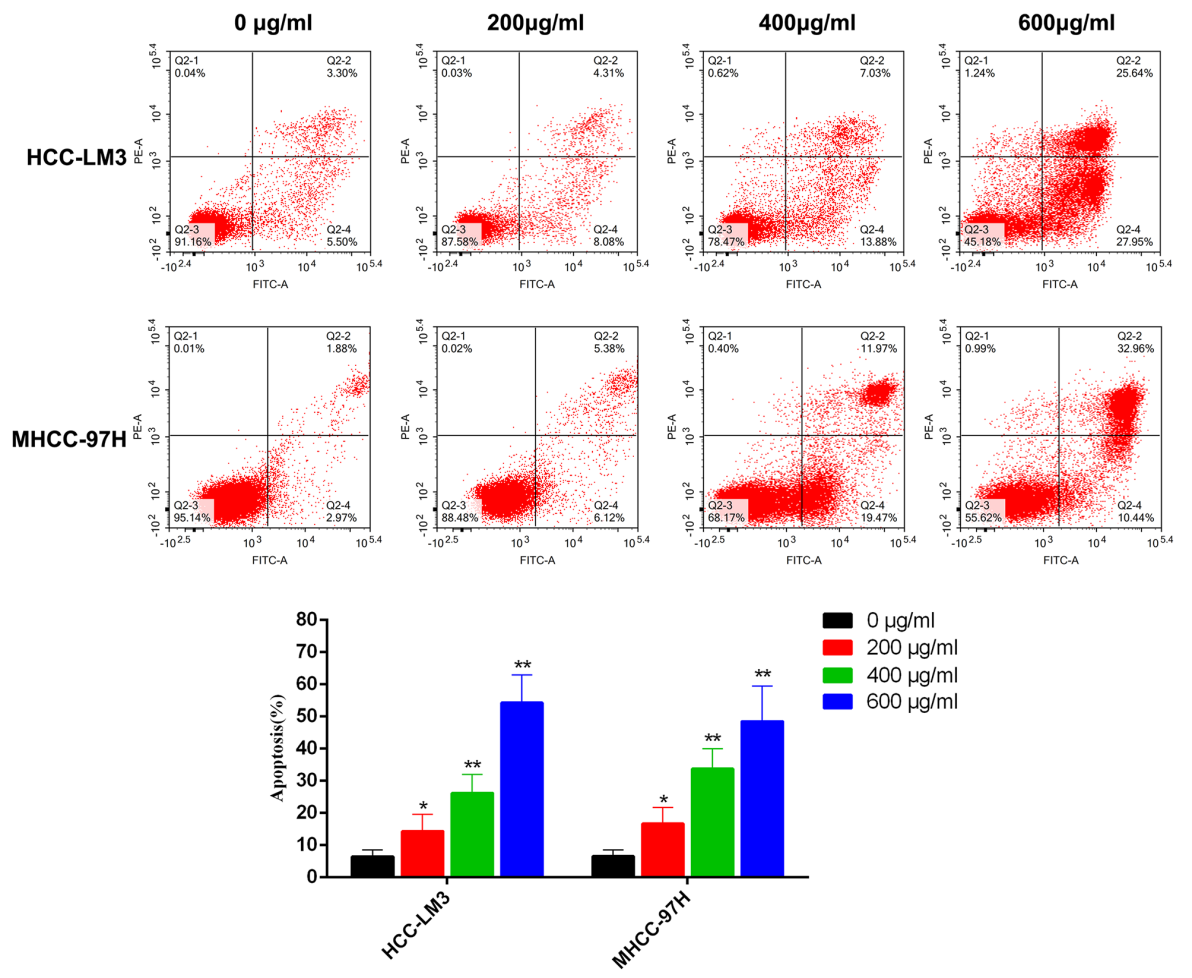
PD promotes the apoptosis of HCC cells and inhibits the proliferation of HCC orthotopic tumors in mice. HCC cells were treated with different concentrations of PD for 48 h, and the apoptosis of HCC cells was detected by flow cytometry. The percentage of apoptotic cells was expressed as the mean ± SD of three independent experiments. *P < 0.05, **P < 0.01 versus the control group

Cell viability experiments showed that PD could induce the death of HCC cells. Therefore, the role of PD on HCC-LM3 and MHCC-97 H cell apoptosis was assessed using flow cytometry. Increasing the concentration of PD increased the apoptosis rate of HCC cells, reaching the maximum at 600 µg/ml. Moreover, apoptosis mainly comprised early apoptosis and late apoptosis (Fig. 7, Supplementary Fig. 2B). These findings demonstrate that PD can induce apoptosis in HCC cells; however, the specific mechanism requires further elucidation.

### PD inhibits PI3K/AKT, TNF, and IL-17 signaling pathways in HCC cells

**Fig. 8:**
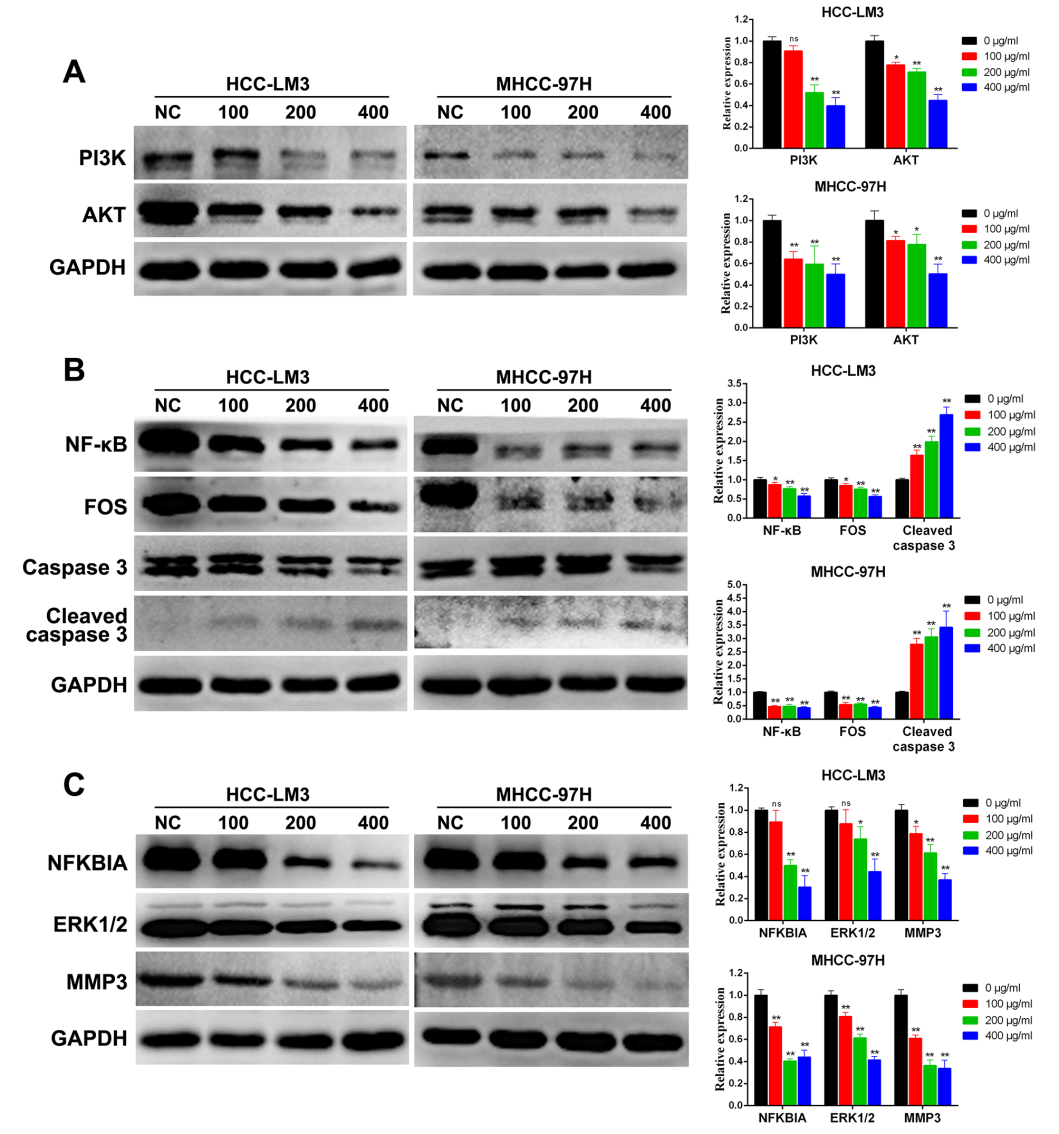
Changes in protein expression of HCC cells treated with PD at different concentrations. (**A**) PI3K/AKT signaling pathway. (**B**) TNF signaling pathway. (**C**) IL-17 signaling pathway. *P < 0.05, **P < 0.01 versus the control group

We further analyzed the potential molecular mechanisms underlying the effect of PD and signaling pathways of PD in HCC treatment using western blotting: PI3K/AKT, TNF, and IL-17 pathway. As shown in Fig. 8A, PI3K and AKT proteins in the PI3K/AKT pathways were significantly downregulated following PD treatment. Moreover, NF-κB and FOS proteins in the TNF pathway were down-regulated, whereas cleaved caspase 3 was upregulated following PD treatment (Fig. 8B). With regard to the IL-17 pathway, the levels of NFKBIA, ERK1/2, and MMP3 proteins were significantly downregulated following PD treatment (Fig. 8C). The results revealed that PI3K/AKT, TNF, and IL-17 signaling pathways play a key role in tumor proliferation and that PD may affect the activation of these pathways to inhibit the proliferation of HCC cells. However, further studies are needed to validate these findings.

## Discussion

The global prevalence of HCC is rising; however, its prognosis remains poor [4, 22]. As liver cancer is not easily detected during the early stages, patients often present with advanced disease. Moreover, there are limited effective treatments for advanced HCC [5]. Recently, TCM is garnering increasing attention for its obvious pharmacological effects and low side effects. Specifically, TCM compounds may have potential applications in the treatment of cancer because they contain several effective active ingredients and have excellent anticancer effects. Therefore, it is essential to analyze the role of TCM in cancer. Several tools and methods have been developed to assess the role of treatments in diseases, particularly cancer. Network pharmacology is a discipline that studies the relationship between drugs and disease targets and is being widely used to analyze and predict new drug targets [23].

PD is a TCM compound that has been proven to have effects in ulcerative colitis [11, 24, 25], Crohn’s disease [26], and the immunogenic death of colorectal cancer cells in combination with 5-FU [10]. PD has been used in Chinese medicine for thousands of years to treat diseases caused by bacteria. The primary active ingredient of PD, *Radix Pulsatillae*, mainly contains [27] saponins [27–29]. The saponin pulchinenoside C is the main effective ingredient of *Pulsatilla*. Moreover, pulchinenoside D is well known for its remarkable anticancer activity against H22 cells [30], HeLa cells [31], and colon cancer [32]. Reportedly, *Pulsatilla* saponins can promote tumor cell death. Xue et al. [33] reported that pulchinenoside C can induce tumor cell apoptosis via the Bcl-2-caspase-3 pathway. Furthermore, pulchinenoside D can inhibit the PI3K–Akt signaling pathway to inhibit tumor growth and induce apoptosis [34]. *Cortex Phellodendri*, a herbal medicine, has been used in China for more than 1,000 years to treat dysentery, jaundice, urinary tract infections, and rheumatoid arthritis [35]. However, it is receiving increasing attention for the prevention of prostate cancer and osteoarthritis. Limonin, extracted from *Cortex Phellodendri*, has been proven to have significant anticancer efficacy [36], particularly by inducing apoptosis in human colon cancer cells [37, 38] and liver cancer cells [39]. In addition, limonin can inhibit glycolysis in liver cancer cells, thus inducing apoptosis [40]. *Rhizoma Coptidis* contains coptisine as its main active ingredient [41]. Coptisine has anticancer, anti-inflammatory, coronary artery disease (CAD) improvement, and antibacterial potential [41]. In addition, it has been found to downregulate the expression of PI3K and AKT and induce the apoptosis of colon cancer cells [42]. Similarly, the anticancer effects of coptisine have been observed in liver, breast, pancreatic, and lung cancers [43–45]. *Cortex Fraxini* is a commonly used TCM compound that has been found to have disease-resistant anti-microbial, anti-tumor, and vascular protection functions [46]. These results indicate that the main active ingredients of PD have anti-tumor effects. However, the antitumor efficacy of PD and the mechanism underlying this effect have not been studied yet.

Although researchers have assessed the antitumor efficacy of the individual components of PD, their efficacy has been limited. The present study is novel as it systematically analyzed the anticancer components of PD through network pharmacology to further study the mechanism and potential targets of PD for the treatment of HCC. Network pharmacology is a new research technology integrating systems biology, bioinformatics, and network science. It can analyze the molecular relationship between drugs and treatment objects from the perspective of the system and the whole biological network to reveal the systematic pharmacological mechanism of drugs [14]. Network pharmacology involves drug targets, disease targets, and their pharmacological relationships and can be used to systematically study the effects of drugs on complex diseases. It can also be used to evaluate the drug compound–target–disease network, which is a novel way to study the mechanism of action of TCM compounds.

Tumor occurrence and development are often characterized by the induction of signaling pathways such as proliferation and invasion. As a result, these signaling pathways are promising targets for cancer treatment [47]. The present study identified 266 compounds, of which 65 active components were identified through network pharmacology analysis. These included 11 compounds in *Radix Pulsatillae*, 37 in *Cortex Phellodendri*, 14 in *Rhizoma Coptidis*, and 3 in *Cortex Fraxini*. Of the 6310 HCC-related genes identified from GeneCards and OMIM, 152 common genes were selected for further analysis. Using PPI network analysis, the potential target genes of PD for the treatment of liver cancer were screened. Furthermore, the active ingredients and molecular mechanisms of PD were analyzed in the treatment of HCC.

The analysis demonstrated that the PI3K/AKT, TNF, and IL-17 pathways may be the key signaling pathways via which PD induces its effect in HCC. AKT signaling has been shown to promote tumor proliferation and metastasis. In addition, the PI3K/AKT signaling pathway can promote the growth and metastasis of tumor cells [48–50]. It additionally regulates cell differentiation, proliferation, migration, and apoptosis by inhibiting or activating a series of downstream substrates through phosphorylation [51, 52]. Alternatively, the TNF signaling pathway was also activated, as revealed by network pharmacology. TNF has been reported to trigger the activation of several pathways, including the NF-κB and MAPK signaling. Western blotting revealed that the expression of NF-κB and FOS was downregulated whereas that of Caspase 3 was upregulated. Reportedly, the upregulation of the NF-κB signaling pathway is related to tumor proliferation and metastasis [53]. NF-κB has anti-apoptotic and pro-angiogenic functions in many cancers [54]. In addition, FOS is a proto-oncogene that promotes tumor proliferation and metastasis [55]. Therefore, NF-κB may be a key molecule that mediates the anticancer effect of PD in HCC, by regulating the proliferation and apoptosis of HCC. The study further revealed that PD treatment downregulated the expression of NFKBIA, ERK1/2, and MMP3. As a member of the MAPK signaling pathway, ERK1/2 can promote the proliferation and differentiation of tumor cells, leading to poor prognosis. Specifically, MMP3 is upregulated in malignant tumors and promotes tumor invasion and metastasis [56]. Therefore, PD can downregulate the expression of NFKBIA, ERK1/2, and MMP3, thus inhibiting the proliferation of HCC and promoting apoptosis. Moreover, IL-17 and TNF signaling pathways are primarily involved in inflammation, tumor cell proliferation, and immune regulation. Dong et al. have demonstrated that downregulating IL17 mRNA levels inhibited the proliferation of tumor cells and reduced the expression of inflammatory cytokines [57]. Moreover, Xun et al. have found that the IL-17 and TNF signaling pathways regulate the expression of PD-L1 in tumor cells, thus regulating the efficacy of drug therapy for tumors [58, 59]. Thus, PD may exert its anti-HCC effects via several pathways, including tumor proliferation, apoptotic, and inflammatory pathways. In addition, many other signaling pathways have been discovered and their role in PD-mediated treatment of HCC needs to be further explored. In conclusion, network pharmacology can effectively predict the antitumor effect of PD.

## Conclusion

Based on network pharmacological analysis, this study confirmed the antitumor efficacy of PD components, which was further validated by in vitro assays. In addition, the study revealed the pathways involved in mediating the anti-HCC effects of PD. Further in vitro experiments confirmed that PI3K/AKT, TNF, and IL-17 pathways mediated the apoptotic effect of PD in HCC cells. These results clarify the mechanism of PD in the treatment of HCC and provide a new therapeutic potential for the treatment of malignant tumors with TCM.

### Electronic supplementary material

Below is the link to the electronic supplementary material.

Supplementary Material 1

Supplementary Material 2

Supplementary Material 3

## Data Availability

The datasets used and/or analyzed during the current study available from the corresponding author on reasonable request.
