# Supplementary material for: Mechanism underlying the effect of Pulsatilla decoction in hepatocellular carcinoma treatment: a network pharmacology and in vitro analysis
Source: BMC Complement Med Ther. 2023 Nov 10;23:405. doi: 10.1186/s12906-023-04244-w (PMC10636957; doi:10.1186/s12906-023-04244-w)
Supplement: Supplementary file 1 — Supplementary Material 1 [file 12906_2023_4244_MOESM1_ESM.pdf]

Supplementary Figure 1

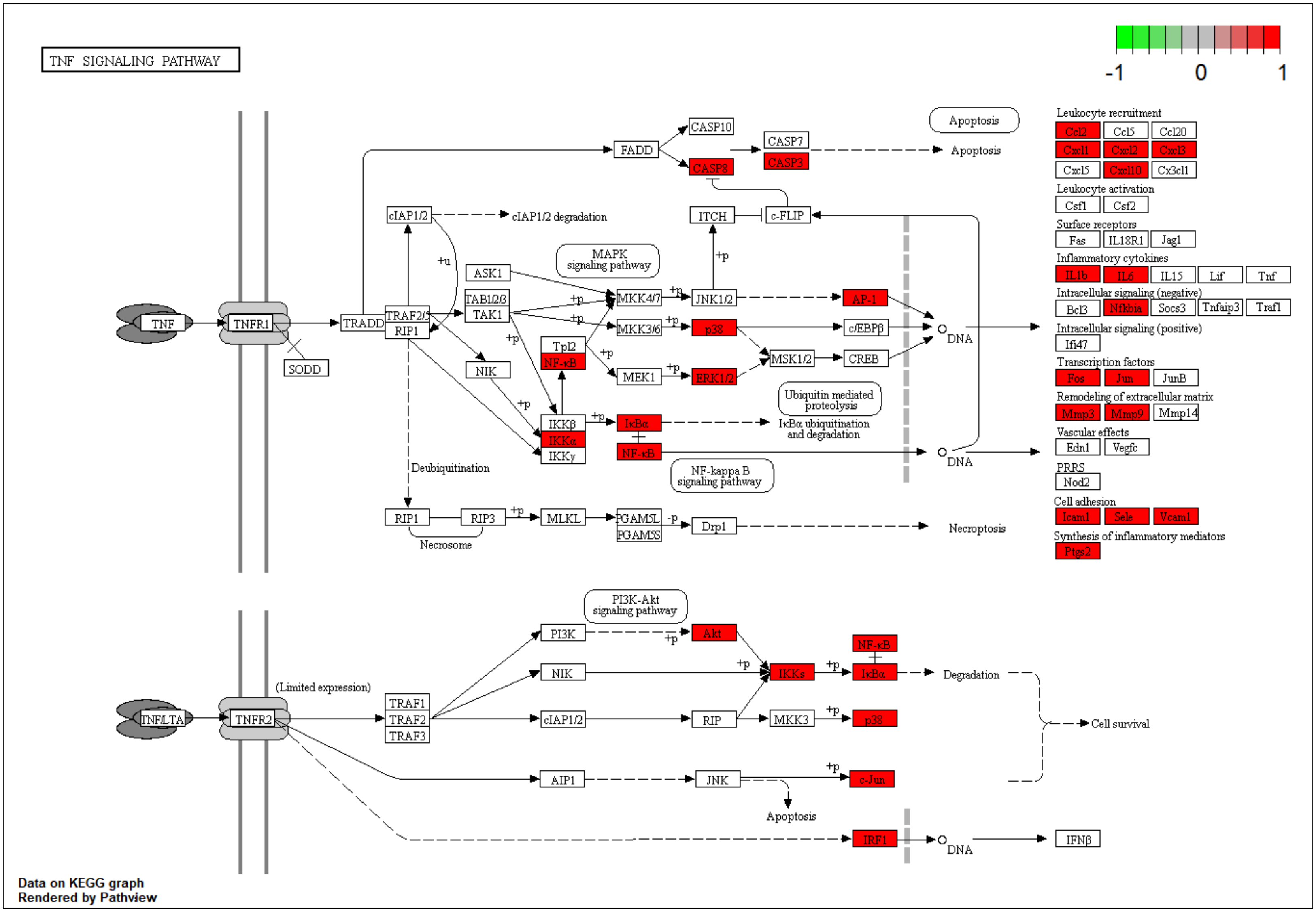

Supplementary Figure 1: The TNF signaling pathway. The TNF signaling pathway may play an important role in the treatment of HCC[1-3].

References:
1. Kanehisa M. Toward understanding the origin and evolution of cellular organisms. Protein Sci. 2019;28(11):1947-51.
2.Kanehisa M, Goto S. KEGG: kyoto encyclopedia of genes and genomes. Nucleic Acids Res. 2000;28(1):27-30.
3.Kanehisa M, Furumichi M, Sato Y, Kawashima M, Ishiguro-Watanabe M. KEGG for taxonomy-based analysis of pathways and genomes. Nucleic Acids Res. 2023;51(D1):D587-d92.
