# Supplementary material for: Mechanism underlying the effect of Pulsatilla decoction in hepatocellular carcinoma treatment: a network pharmacology and in vitro analysis
Source: BMC Complement Med Ther. 2023 Nov 10;23:405. doi: 10.1186/s12906-023-04244-w (PMC10636957; doi:10.1186/s12906-023-04244-w)
Supplement: Supplementary file 2 — Supplementary Material 2 [file 12906_2023_4244_MOESM2_ESM.pdf]

Supplementary Figure 2

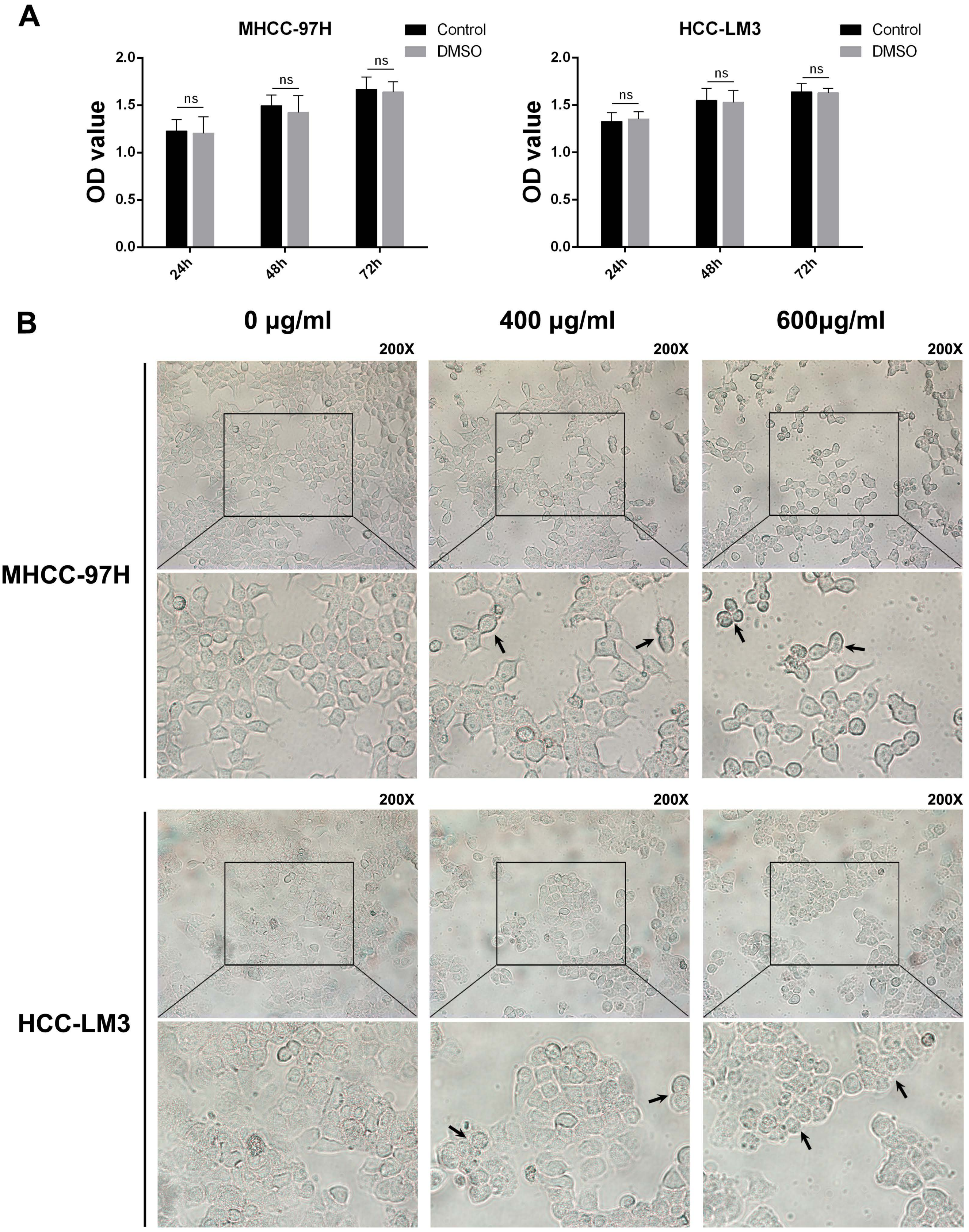

Supplementary Figure 2: DMSO toxicity assays and microscopic images of apoptosis after PD treatment. (A)OD values of cell viability of HCC-LM3 and MHCC-97H cell lines incubated with DMSO (0.5%) for 24h, 48h and 72h, respectively. ns=no significant versus the untreated group. (B) Micrographs of apoptosis of MHCC-97H and HCC-LM3 cell lines treated with different concentrations of PD (shown by black arrows).
