# Supplementary material for: Mechanism underlying the effect of Pulsatilla decoction in hepatocellular carcinoma treatment: a network pharmacology and in vitro analysis
Source: BMC Complement Med Ther. 2023 Nov 10;23:405. doi: 10.1186/s12906-023-04244-w (PMC10636957; doi:10.1186/s12906-023-04244-w)
Supplement: Supplementary file 3 — Supplementary Material 3 [file 12906_2023_4244_MOESM3_ESM.pdf]

Raw data of Figure 8：

(The first experiment)

Figure 8A：

	HCC-LM3	MHCC-97H
PI3K (110kDa)	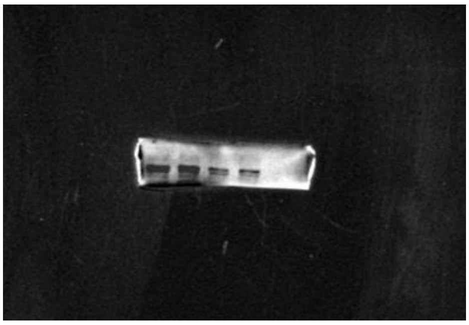	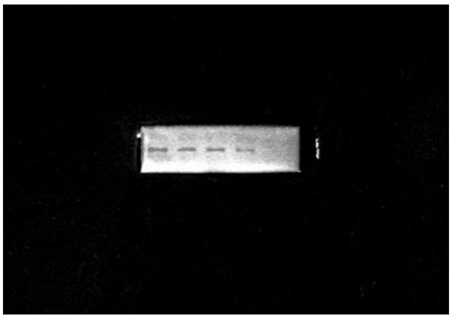
AKT (60kDa)	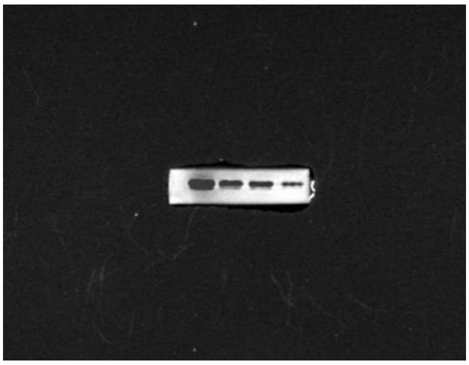	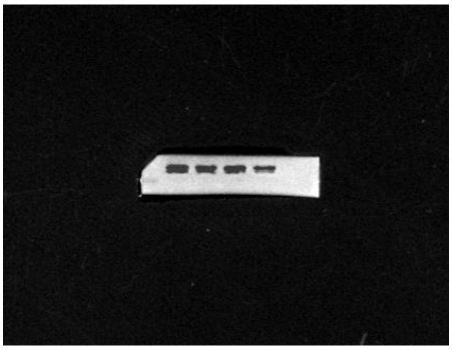
GAPDH (36 kDa)	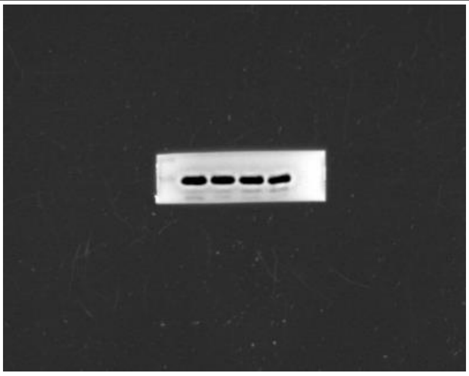	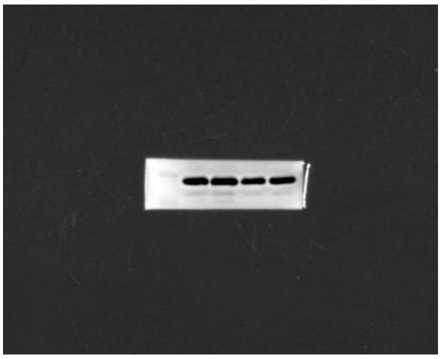

Figure 8B：

	HCC-LM3	MHCC-97H
NF-κB (50kDa)	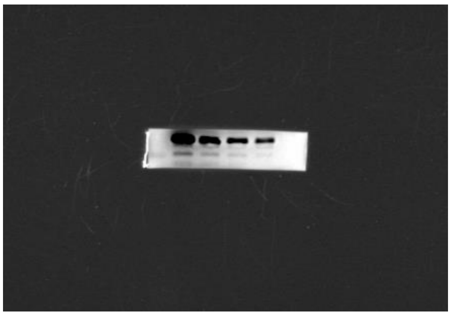	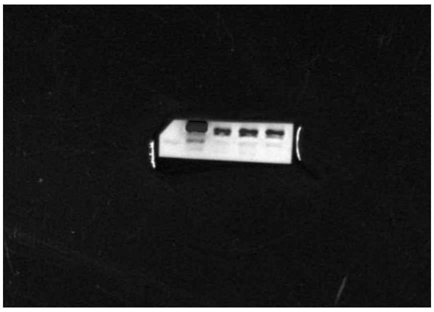
FOS (55-60 kDa)	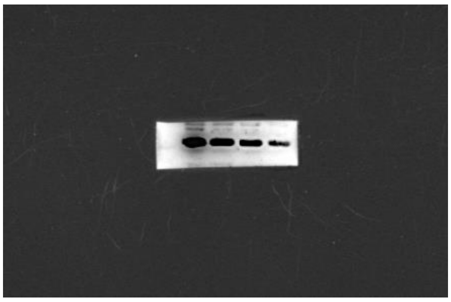	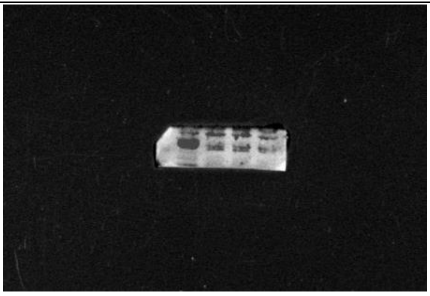
Caspase 3 (32 kDa)	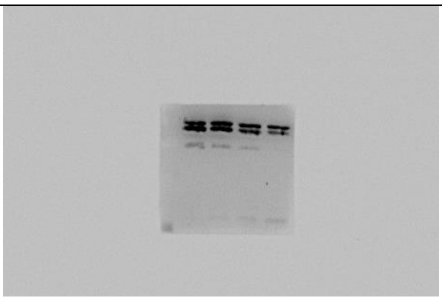	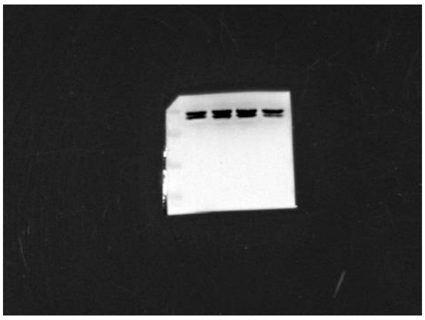
Cleaved Caspase 3 (17 kDa)	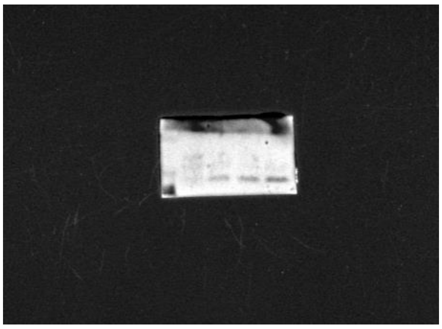	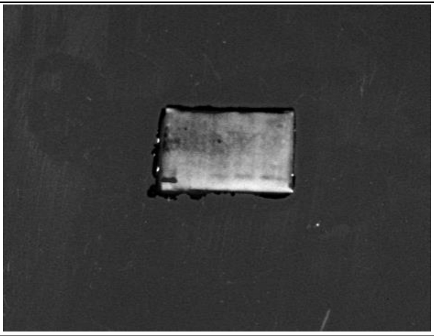
GADPH (36 kDa)	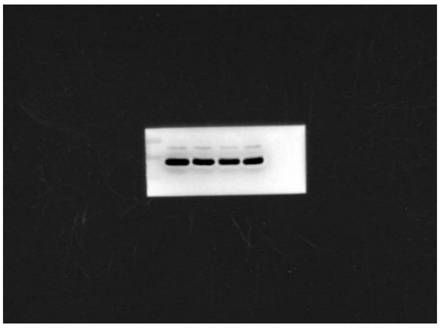	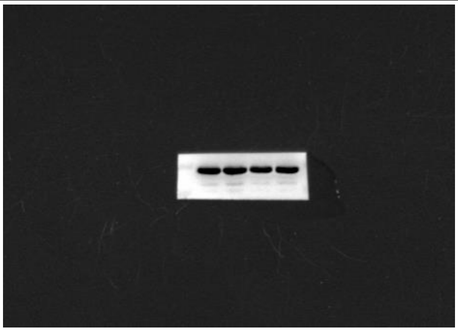

Figure 8C：

	HCC-LM3	MHCC-97H
NFKBIA (36kDa)	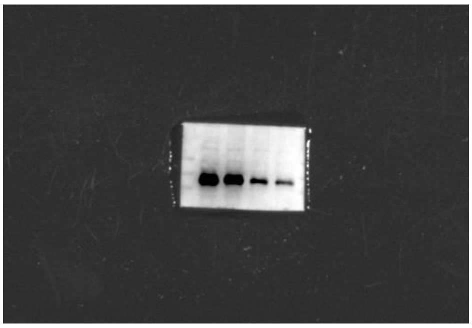	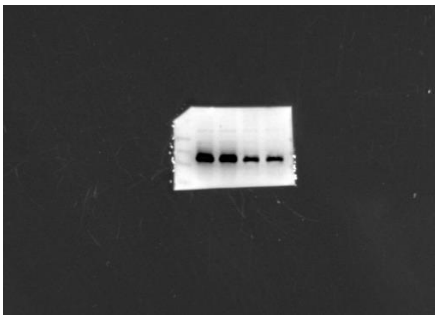
ERK1/2 (38-44 kDa)	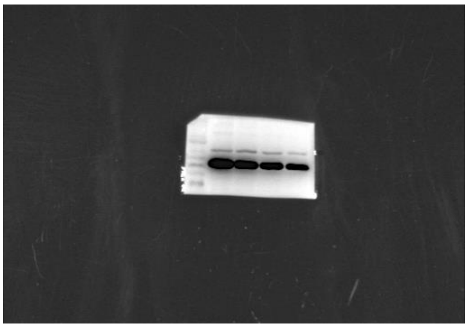	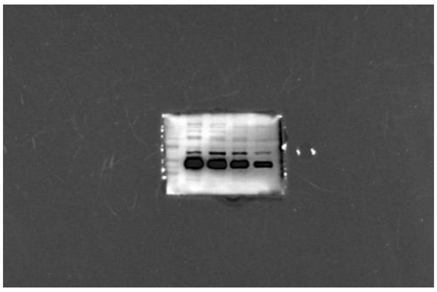
MMP3 (45-60 kDa)	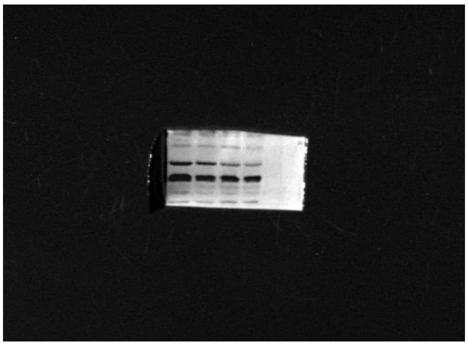	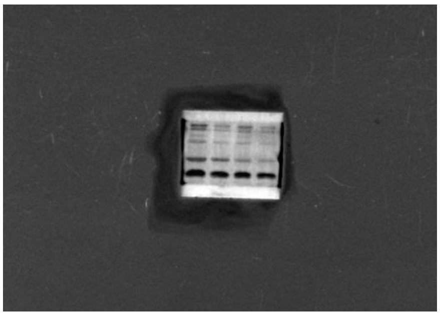
GADPH (36 kDa)	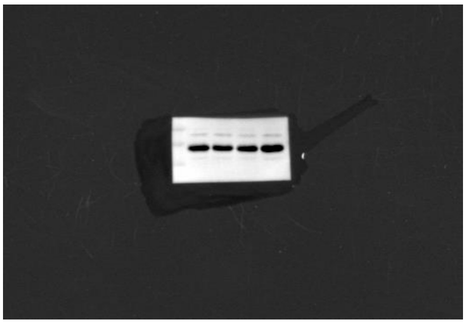	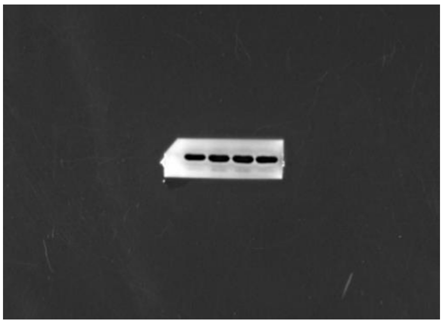

Raw data of Figure 8
(The second experiment)

Figure 8A：

	HCC-LM3	MHCC-97H
PI3K (110kDa)	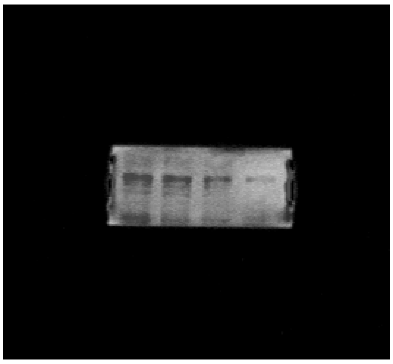	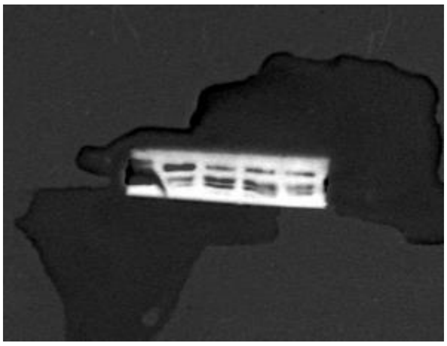
AKT (60kDa)	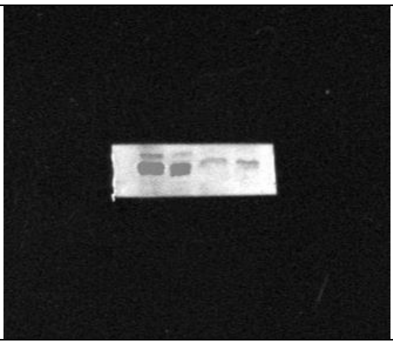	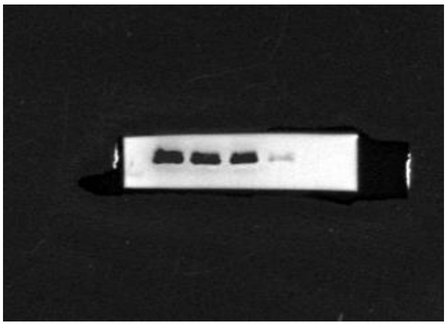
GAPDH (36 kDa)	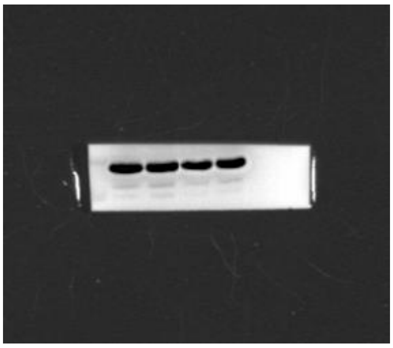	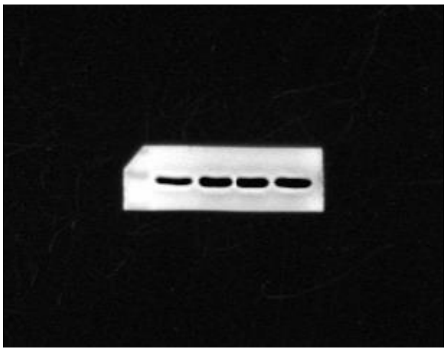

Figure 8B：

	HCC-LM3	MHCC-97H
NF-κB (50kDa)	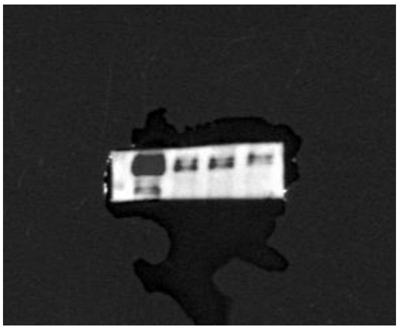	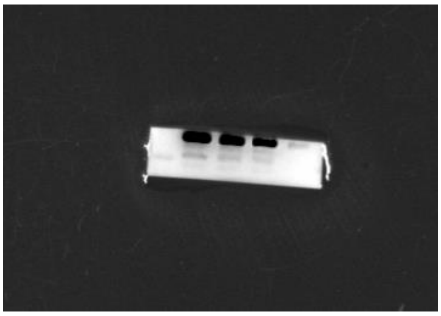
FOS (55-60 kDa)	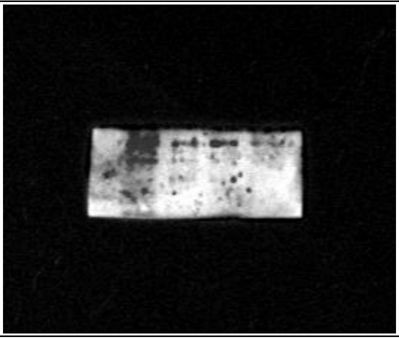	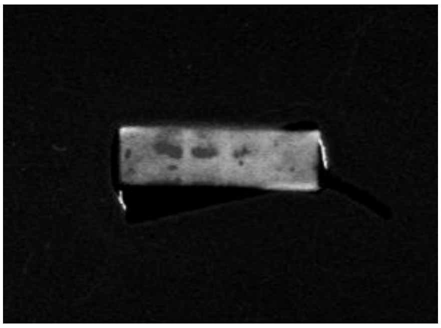
Caspase 3 (32 kDa)	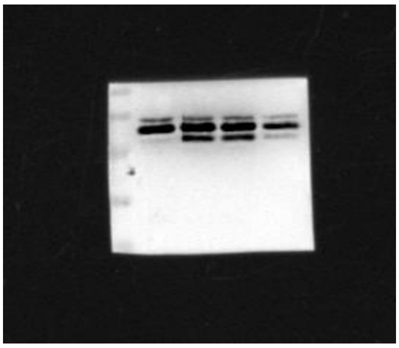	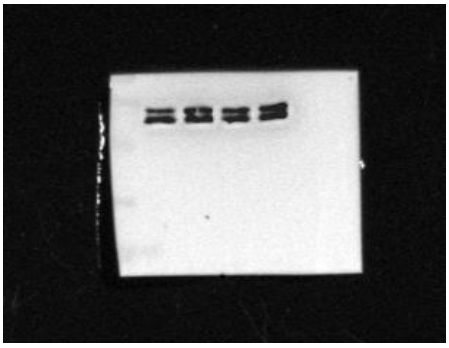
Cleaved Caspase 3 (17 kDa)	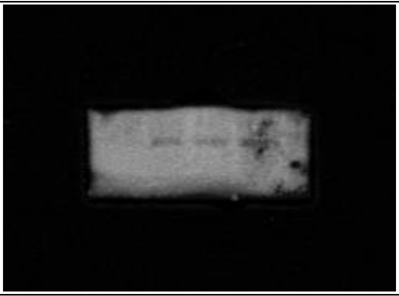	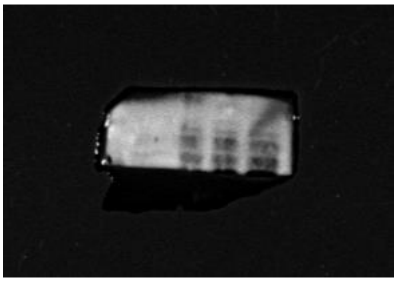
GADPH (36 kDa)	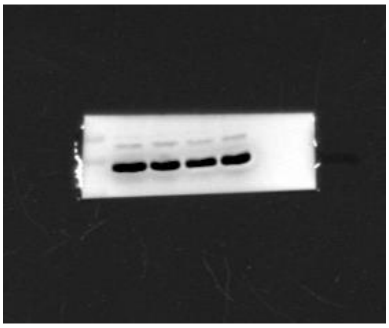	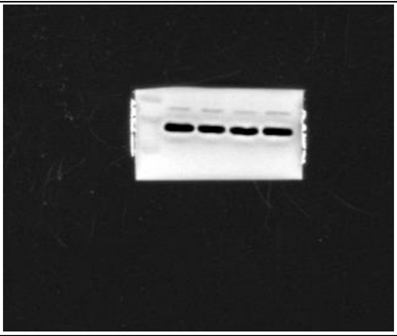

Figure 8C：

	HCC-LM3	MHCC-97H
NFKBIA<br>(36kDa)	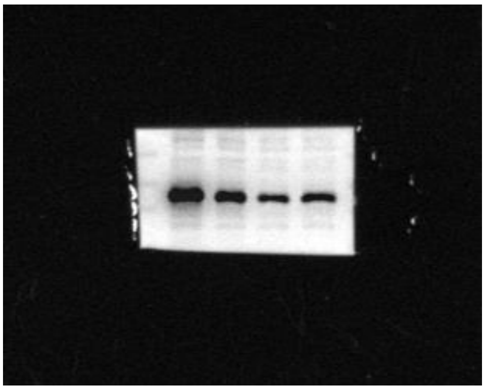	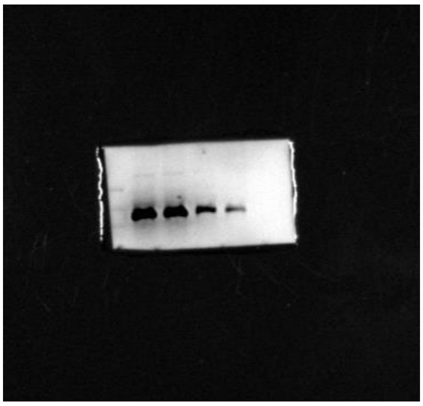
ERK1/2<br>(38-44<br>kDa)	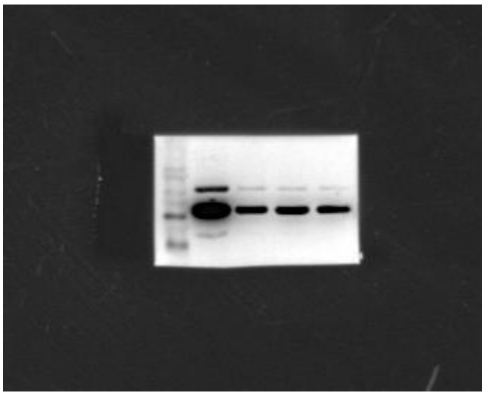	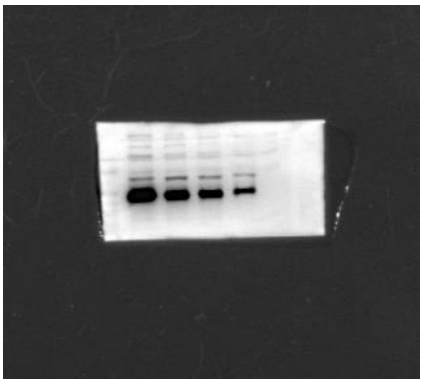
MMP3<br>(45-60<br>kDa)	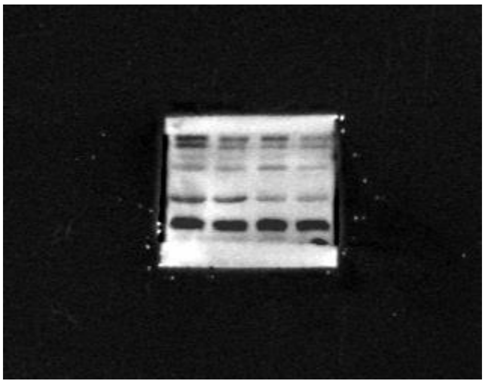	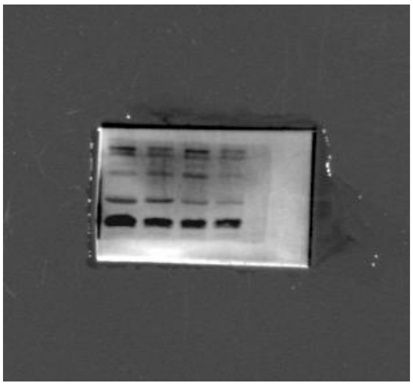
GADPH<br>(36 kDa)	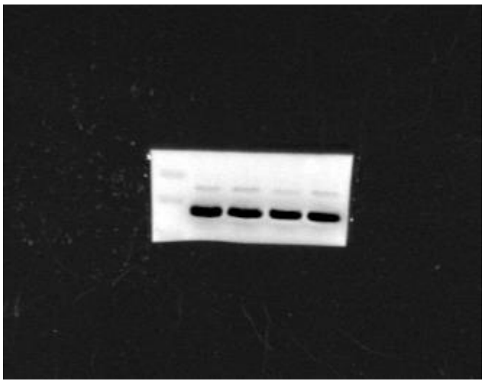	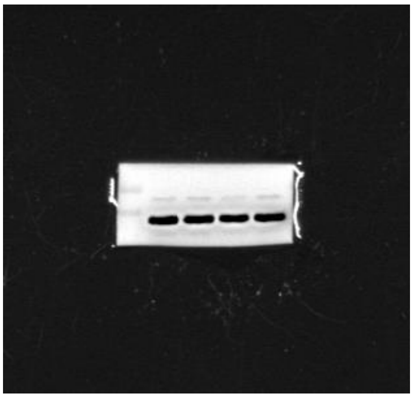

Raw data of Figure 8: Changes in protein expression of HCC cells treated with PD at different concentrations. This supplementary Figure is the raw data of Figure 8.
